# Identification of a new allele of *O-fucosyltransferase 1* involved in *Drosophila* intestinal stem cell regulation

**DOI:** 10.1242/bio.058910

**Published:** 2021-11-03

**Authors:** Lin Shi, Ruiyan Kong, Zhengran Li, Hang Zhao, Rui Ma, Guang Bai, Jing Li, Zhouhua Li

**Affiliations:** 1 College of Life Sciences, Capital Normal University, Beijing 100048, China; 2 Department of Neurology, Xuanwu Hospital, Capital Medical University, Beijing 100053, China

**Keywords:** O-fut1, Intestinal stem cell, *Drosophila*, Notch signaling

## Abstract

Adult stem cells are critical for the maintenance of tissue homeostasis. However, how the proliferation and differentiation of intestinal stem cells (ISCs) are regulated remains not fully understood. Here, we find a mutant, *stum 9-3*, affecting the proliferation and differentiation of *Drosophila* adult ISCs in a forward genetic screen for factors regulating the proliferation and differentiation ISCs. *stum 9-3* acts through the conserved Notch signaling pathway, upstream of the S2 cleavage of the Notch receptor. Interestingly, the phenotype of *stum 9-3* mutant is not caused by disruption of *stumble* (*stum*), where the p-element is inserted. Detailed mapping, rescue experiments and mutant characterization show that *stum 9-3* is a new allele of *O-fucosyltransferase 1* (*O-fut1*). Our results indicate that unexpected mutants with interesting phenotype could be recovered in forward genetic screens using known p-element insertion stocks.

## Abstract (precis)

**Summary:** A mutant, *stum 9-3*, affecting the proliferation and differentiation of *Drosophila* adult intestinal stem cells (ISCs) was identified in a forward genetic screen for factors regulating the proliferation and differentiation ISCs. *stum 9-3* acts through the Notch signaling pathway. Detailed mapping, rescue experiments and characterization show that *stum 9-3* is not a *stumble* mutant where the p-element is inserted, but a new allele of *O-fucosyltransferase 1* (*O-fut1*).

## INTRODUCTION

Stem cells are responsible for maintaining the homeostasis of adult tissues, where the frequently lost cells are constantly replenished by stem cell progeny. The proliferation and differentiation of adult stem cells must be tightly controlled. Disruption of this balance will lead to stem cell accumulation or stem cell depletion, eventually resulting in various diseases, such as cancer and aging (Jasper, 2020; Lin, 2008; Morrison and Spradling, 2008; Radtke and Clevers, 2005; Singh et al., 2019). Therefore, understanding of the mechanisms controlling stem cell proliferation and differentiation will provide insight into the development of therapeutics to treat human diseases.

The posterior midgut of the adult *Drosophila* intestine has proven to be an excellent system to study the regulation of stem cell proliferation and differentiation. *Drosophila* intestines show marked similarities with their mammalian counterparts in terms of cellular make-up, development and genetic control (Banerjee et al., 2019; Casali and Batlle, 2009; Gervais and Bardin, 2017; Jasper, 2020; Stainier, 2005; Wang and Hou, 2010). ISCs are distributed along the basement membrane of the *Drosophila* adult midgut epithelium (Micchelli and Perrimon, 2006; Ohlstein and Spradling, 2006). Initial studies proposed that ISCs constantly undergo asymmetric divisions and produce non-dividing daughter cells, enteroblasts (EBs) (Micchelli and Perrimon, 2006; Ohlstein and Spradling, 2006, 2007). Delta (Dl), one of the Notch (N) ligands, is specifically expressed in ISCs, while Notch receptor is expressed in both ISCs and EBs. ISCs signal via Dl to activate Notch signaling in EBs, which terminally differentiate into either absorptive enterocytes (ECs) or secretory enteroendocrine cells (EE) depending on their signaling environments (Beebe et al., 2010; Micchelli and Perrimon, 2006; Ohlstein and Spradling, 2006; Perdigoto et al., 2011; Yeung et al., 2011). Recent studies show that a significant proportion of ISCs divides symmetrically in response to differentiation and subsequent loss of a neighboring ISC (or vice versa) (de Navascués et al., 2012; Goulas et al., 2012; O'Brien et al., 2011). Moreover, EE cells may not be generated from EBs, but directly from ISCs or EE progenitor cells (EEPs) (Biteau and Jasper, 2014; Chen et al., 2018; Zeng et al., 2015). Interestingly, unlike other systems in which differentiated cells could be de-differentiated into stem cells, no new ISCs could be re-generated after all progenitors were ablated, indicating that fully differentiated intestinal cells could not de-differentiate into ISCs in the absence of any progenitors (Brawley and Matunis, 2004; Lu and Li, 2015a; Raff, 2003).

Previous studies have shown that the proliferation and differentiation of ISCs under physiological and stressed conditions are regulated by many signaling pathways including the Notch, Wingless (Wg), Janus Kinase/Signal Transducer and Activator of Transcription (JAK/STAT), Epidermal Growth Factor Receptor (EGFR), Hippo (Hpo), Insulin, Hedgehog (Hh), and Bone Morphogenetic Protein (BMP) pathways (see reviews by Colombani and Andersen, 2020; Gervais and Bardin, 2017; Guo et al., 2016; Jiang et al., 2016; Joly and Rousset, 2020; and references therein). The evolutionarily conserved Notch signaling pathway plays essential roles in the control of cell proliferation and specification/differentiation during animal development (Artavanis-Tsakonas and Muskavitch, 2010). Before being presented on the plasma membrane, the Notch receptor undergoes post-translational modifications such as fucosylation in the ER and Golgi apparatus where the S1 proteolytic cleavage of the Notch receptor occurs. Upon binding of its ligand (Dl/Ser) from the signal sending cell on the plasma membrane, the Notch receptor in the signal receiving cell undergoes two consecutive proteolytic cleavages (S2 and S3 cleavages), producing Notch extracellular truncate (NEXT, the S2 cleavage product) and finally releasing the intracellular domain of Notch (NICD, the S3 cleavage product) from the plasma membrane to regulate the expression of downstream genes in the nucleus (Artavanis-Tsakonas and Muskavitch, 2010; Artavanis-Tsakonas et al., 1999; Bray, 2006; Okajima and Irvine, 2002; Pandey et al., 2021). Notch signaling is essential for the proliferation and differentiation of ISCs in *Drosophila*, loss of Notch signaling in ISCs leads to increased ISC proliferation and progeny differentiation defects, generating ISC and/or EE tumors, while constitutive activation of Notch signaling forces direct differentiation of ISCs into ECs, resulting in ISC loss (Micchelli and Perrimon, 2006; Ohlstein and Spradling, 2006, 2007).

## RESULTS AND DISCUSSION

**Fig. 1.**
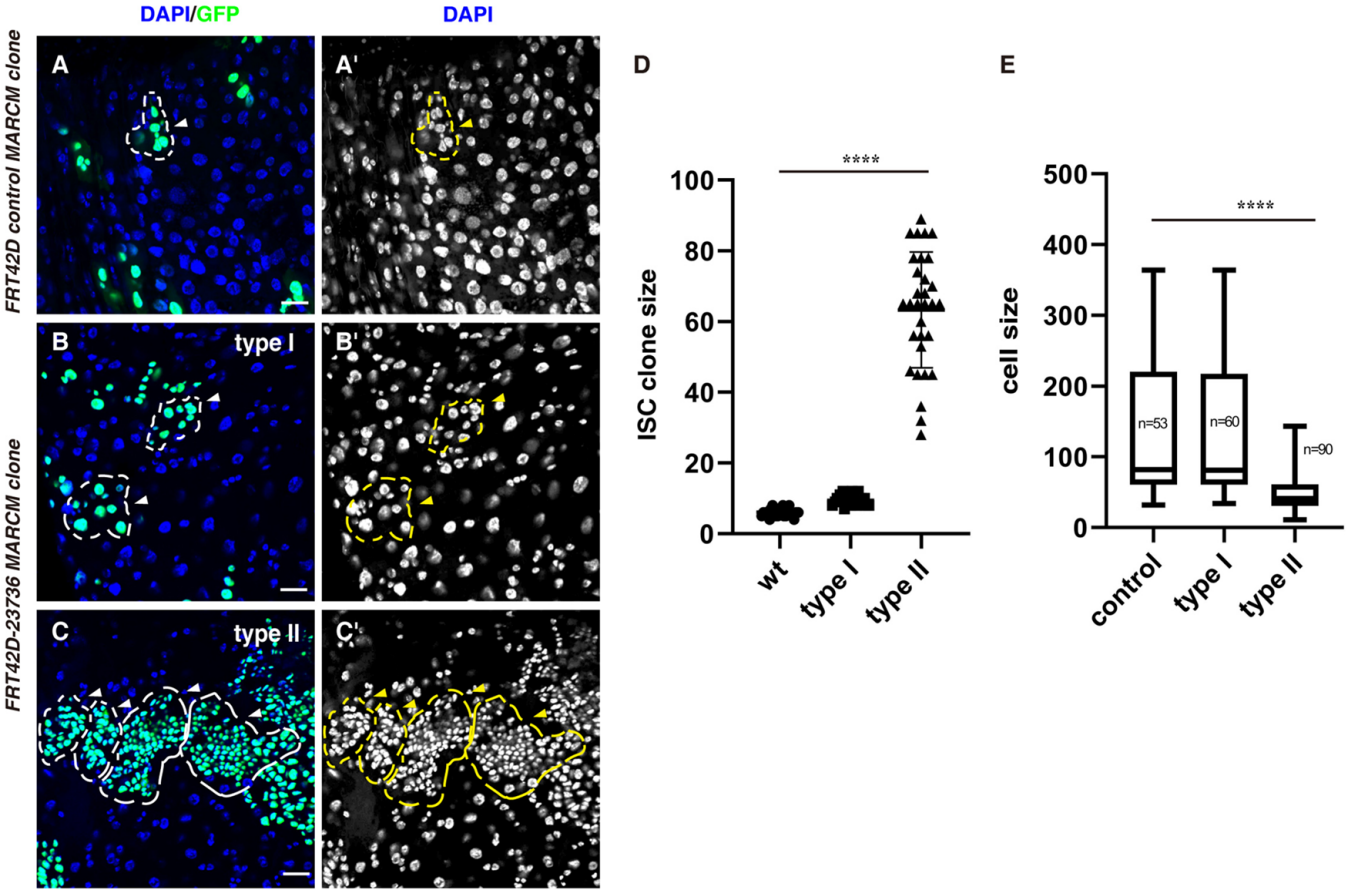
**Two type of ISC clones are observed in *FRT42D-23736* mutant.** (A) *FRT42D* control ISC MARCM *clones* (green) (white arrowhead). One ISC MARCM clone is labeled with dotted lines. DAPI staining for the nucleus is showed separately. (B) Type I ISC MARCM clones from *FRT42D-23736* mutant (green) (white arrowheads). Please note that the size of type I clone is larger than that of control clone. DAPI staining for the nucleus is showed separately. (C) Type II ISC MARCM clones from *FRT42D-23736* mutant (green) (white arrowheads). Please note that the size of type II clone is much larger than that of control and type I clones and the cells in these clones are small and quite uniform in size. DAPI staining for the nucleus is showed separately. (D) Quantification of the size of ISC MARCM clones indicated. Mean±s.d. is shown. *n*=30–35. ****P<0.0001. (E) Quantification of the cell size of ISC MARCM clones indicated. Mean±s.d. is shown. ****P<0.0001. Please note that the type II clones are deformed, preventing accurate quantification of ISC MARCM clone size. Scale bars: 20 μm.

To identify factors regulating the proliferation and differentiation of ISCs, we carried out a small-scale forward genetic screen using some p-element insertions by MARCM technique in the posterior midgut (data not shown) (Lee and Luo, 2001). We were surprised to find that there are two types of ISC MARCAM clones generated upon induction of *stum^MB01421^* mutant (Fig. 1A,B). A p-element is inserted in the coding region of *stumble* (*stum*, *stum^MB01421^*), which is required for locomotion and mechanical sensing in proprioceptive neurons by transducing dendrite stretching into cellular responses (Desai et al., 2014). The type I *stum^MB01421^* ISC MARCM clones are similar as those of wild-type (WT) control (Fig. 1B,D,E). While the type II *stum^MB01421^* ISC MARCM clones grow much faster than those of WT clones, and the cell size in these clones is tiny and quite uniform compared to the varied cell size of control and type I clones, suggesting that ISC proliferation and/or differentiation is affected in these clones (Fig. 1C–E). We purified the mutant for the type II clones by consecutive backcrosses and named this mutant as *stum 9-3* (data not shown).

**Fig. 2.**
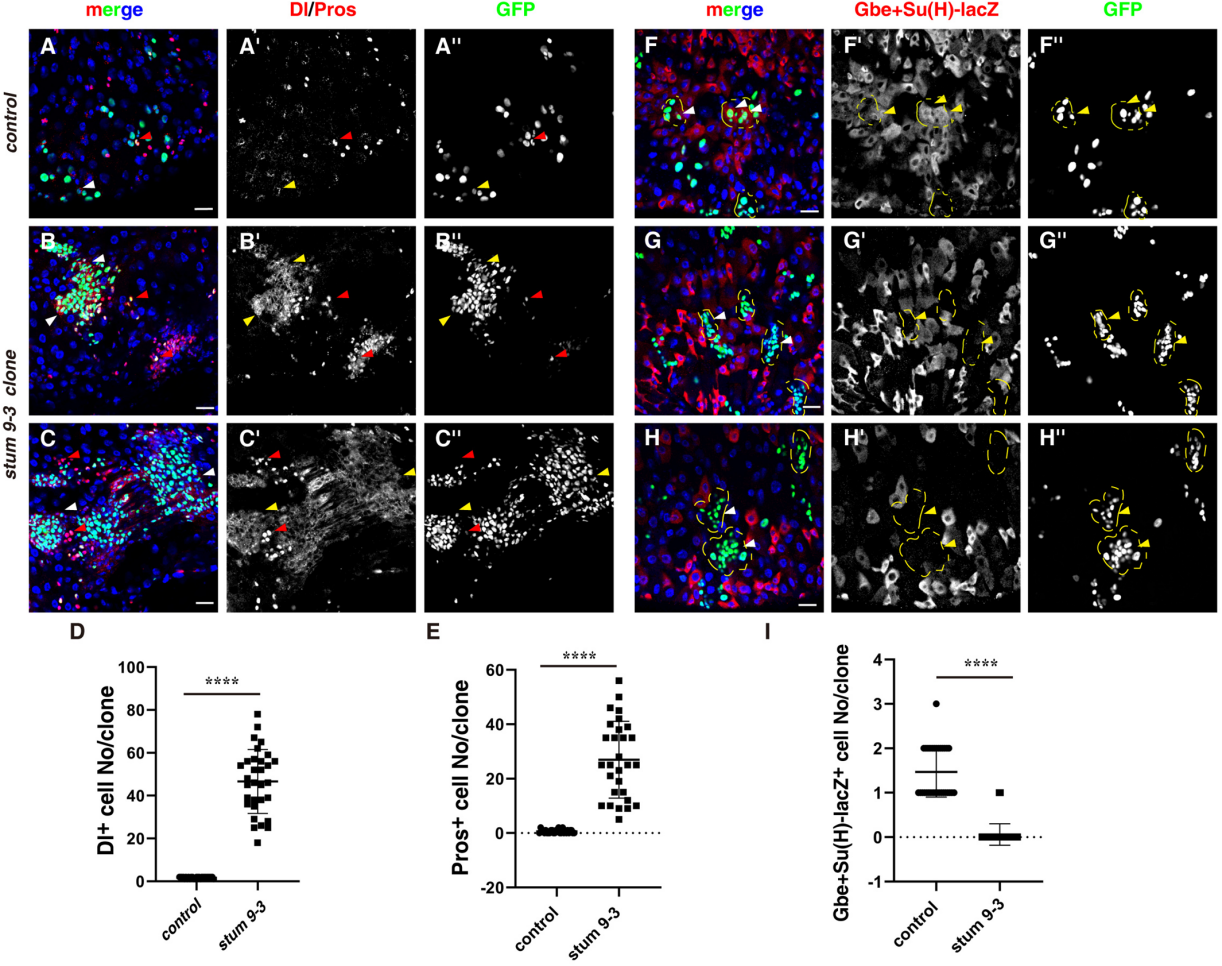
***stum 9-3* affects Notch signaling.** (A) Dl (ISC marker) and Pros (EE marker) (red) in intestines with control MARCM clones (white and red arrowheads). Dl/Pros and GFP channels are showed separately in black-white. Please note that EE cell could be observed in some control ISC clones (red arrowhead). (B,C) Dl and Pros (red) in intestines with *stum 9-3* ISC MARCM clones (white and red arrowheads). Please note that the number of Dl+ and Pros+ cells is dramatically increased. Two types of clones are observed: ISC clones and EE clones. (D) Quantification of the number of Dl+ cells per clone in control and *stum 9-3* intestines. *n*=25–35. Mean±s.d. is shown, ****P<0.0001. Please note that *stum 9-3* ISC clones are deformed, preventing accurate quantification of Dl+ cells per clone. (E) Quantification of the number of EE cells per clone in control and *stum 9-3* intestines. *n*=25–35. Mean±s.d. is shown, ****P<0.0001. Please note that *stum 9-3* ISC clones are deformed, preventing accurate quantification of EE cells per clone. (F) *Gbe+Su(H)-lacZ* cells (Notch signaling reporter, EBs, red) in control ISC MARCM clones (labeled with yellow dotted lines, white arrowheads). Please note that these clones contain several *Gbe+Su(H)-lacZ+* cells, indicating that Notch signaling is activated in these clones. *Gbe+Su(H)-lacZ* and GFP channels are showed separately in black-white. (G,H) *Gbe+Su(H)-lacZ* cells (EBs, red) in *stum 9-3* ISC MARCM clones (with yellow dotted lines, white arrowheads). Please note that no *Gbe+Su(H)-lacZ+* cells are observed in these clones, indicative of defective Notch signaling. (I) Quantification of the number of *Gbe+Su(H)-lacZ*^+^ cells per clone in control and *stum 9-3* intestines. *n*=25–35. Mean±s.d. is shown, ****P<0.0001. GFP is in green, blue indicates DAPI staining for DNA. Scale bars: 20 μm. Yellow arrows correspond to the white arrows, pointing to the cells that the white arrows pointed to.

We examined the cell identity in *stum 9-3* mutant clones. One to two Dl+ cells could be observed in control clones, while the number of Dl+ cells is dramatically increased in *stum 9-3* mutant clones, suggesting that *stum 9-3* mutant affects ISC proliferation and/or differentiation (Fig. 2A–D). Meanwhile, EE cell (Pros+) could be observed in control clones (Fig. 2A). On the contrary, two types of *stum 9-3* mutant clones are observed: the number of EE cells is significantly increased and increased number of EE cells could be observed adjacent to some clones; while the majority of the other type clones are EE cells (Fig. 2B,C,E). These data suggest that *stum 9-3* mutant likely affects ISC proliferation and differentiation. The phenotype of *stum 9-3* mutant is reminiscent of *Notch* loss of function (Fig. S1) (Micchelli and Perrimon, 2006; Ohlstein and Spradling, 2006, 2007). We then examined whether Notch signaling is affected in *stum 9-3* mutant ISC MARCM clones. Notch signaling is activated [by Gbe+Su(H)-lacZ] in control clones (Fig. 2F) (Furriols and Bray, 2001). However, no Gbe+Su(H)-lacZ+ cells are observed in *stum 9-3* ISC clones, indicating that Notch signaling is abolished in *stum 9-3* mutant (Fig. 2G–I). In supporting of this, the expression of Notch downstream target Cut during wing development is also abolished in *stum 9-3* mutant clones, indicating that *stum 9-3* mutant is generally required for the activation of Notch signaling (Fig. S2). Furthermore, we find that both NECD and NICD are highly accumulated in *stum 9-3* ISC MARCM clones, suggesting that the full-length Notch receptor is accumulated in *stum 9-3* mutant clones (Fig. S3). Altogether, these data indicate that *stum 9-3* mutant affects the activation of Notch signaling.

**Fig. 3.**
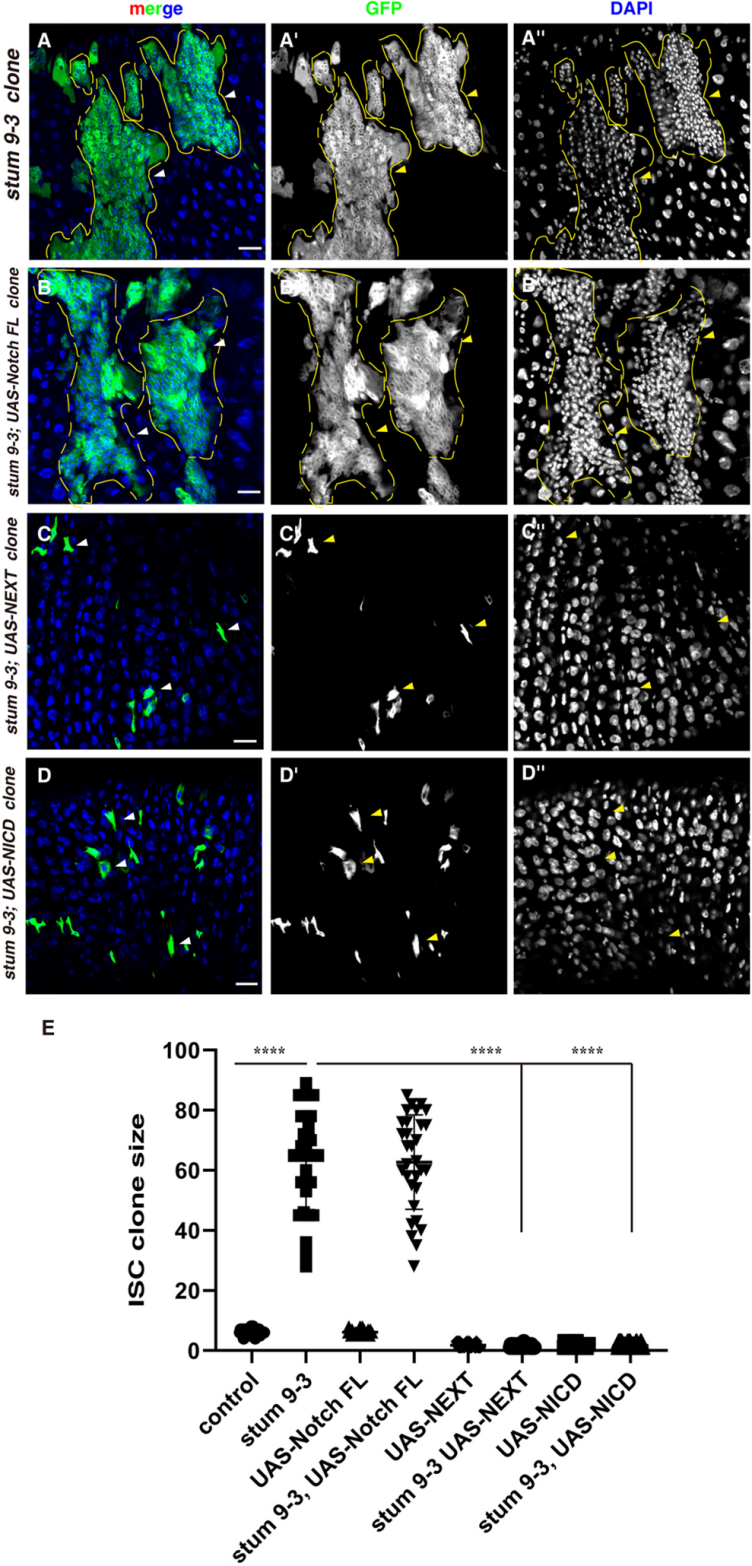
***stum 9-3* functions upstream of the S2 cleavage of the Notch receptor.** (A) *stum 9-3* ISC MARCM clones (green) (labeled with yellow dotted lines, white arrowheads). GFP and DAPI channels are showed separately in black-white. (B) Expression of full-length Notch could not rescue defects observed in *stum 9-3* ISC MARCM clones (green) (labeled with yellow dotted lines, white arrowheads). (C,D) Expression of NEXT (C) or NICD (D) could completely suppress ISC over-proliferation observed in *stum 9-3* ISC MARCM clones (green) (labeled with yellow dotted lines, white arrowheads). (E) Quantification of the size of ISC MARCM clones in different genotypes indicated. *n*=25–35. Mean±s.d. is shown, ****P<0.0001. Please note that *stum 9-3* ISC clones are deformed, preventing accurate quantification of clone size. In all panels except graphs, GFP is in green, blue indicates DAPI staining for DNA. Scale bars: 20 μm. Yellow arrows correspond to the white arrows, pointing to the cells that the white arrows pointed to.

We then examined which step of Notch receptor processing that *stum 9-3* mutant is required for. We performed rescue experiments by expressing full-length Notch (S1 cleavage product), NEXT (the S2 cleavage product) and NICD (the S3 cleavage product) in *stum 9-3* mutant clones, respectively. We found that expression of full-length Notch could not rescue defects observed in *stum 9-3* mutant clones (Fig. 3A,B; Fig. S4). While expression of either NEXT or NICD dramatically reduced the size of *stum 9-3* mutant clones, likely causing precocious ISC differentiation due to ectopic activation of Notch signaling (Fig. 2C–E; Fig. S4) (Micchelli and Perrimon, 2006; Ohlstein and Spradling, 2006). These data suggest that *stum 9-3* mutant affects Notch signaling upstream of the S2 cleavage of the Notch receptor, with accumulation of full-length Notch receptors in *stum 9-3* mutant clones. The retromer complex is reported to directly regulate Notch receptor retrograde trafficking in *Drosophila* neuroblast lineages to prevent aberrant Notch signaling activation (Li et al., 2018). However, depleting the retromer complex could not rescue defects observed in *stum 9-3* mutant clones, indicating that *stum 9-3* mutant may act upstream of endocytic and retrograde trafficking of the Notch receptor (Fig. S5). Taken together, these data show that *stum 9-3* mutant is involved in Notch signaling upstream of the S2 cleavage of the Notch receptor.

As *stum 9-3* mutant is derived from *stum^MB01421^* mutant, which disrupts *stum* function, we then examined whether the defects observed in *stum 9-3* mutant are resulted from *stum* loss of function. However, we found that *stum* is not expressed in the intestines (by *stumGal4*) (Fig. S6A–B′) (Desai et al., 2014). Furthermore, no obvious defects were detected in the adult intestines of homozygous *stum* null mutants (Fig. S6C,D) (Desai et al., 2014). Moreover, GFP-Stum is localized in the cell periphery when expressed in the progenitors and expression of *GFP-stum* could not rescue defects observed in *stum 9-3* mutant (Fig. S6E–J) (Desai et al., 2014). Altogether, these data demonstrate that the defects observed in *stum 9-3* mutant are not caused by *stum* loss of function.

**Fig. 4.**
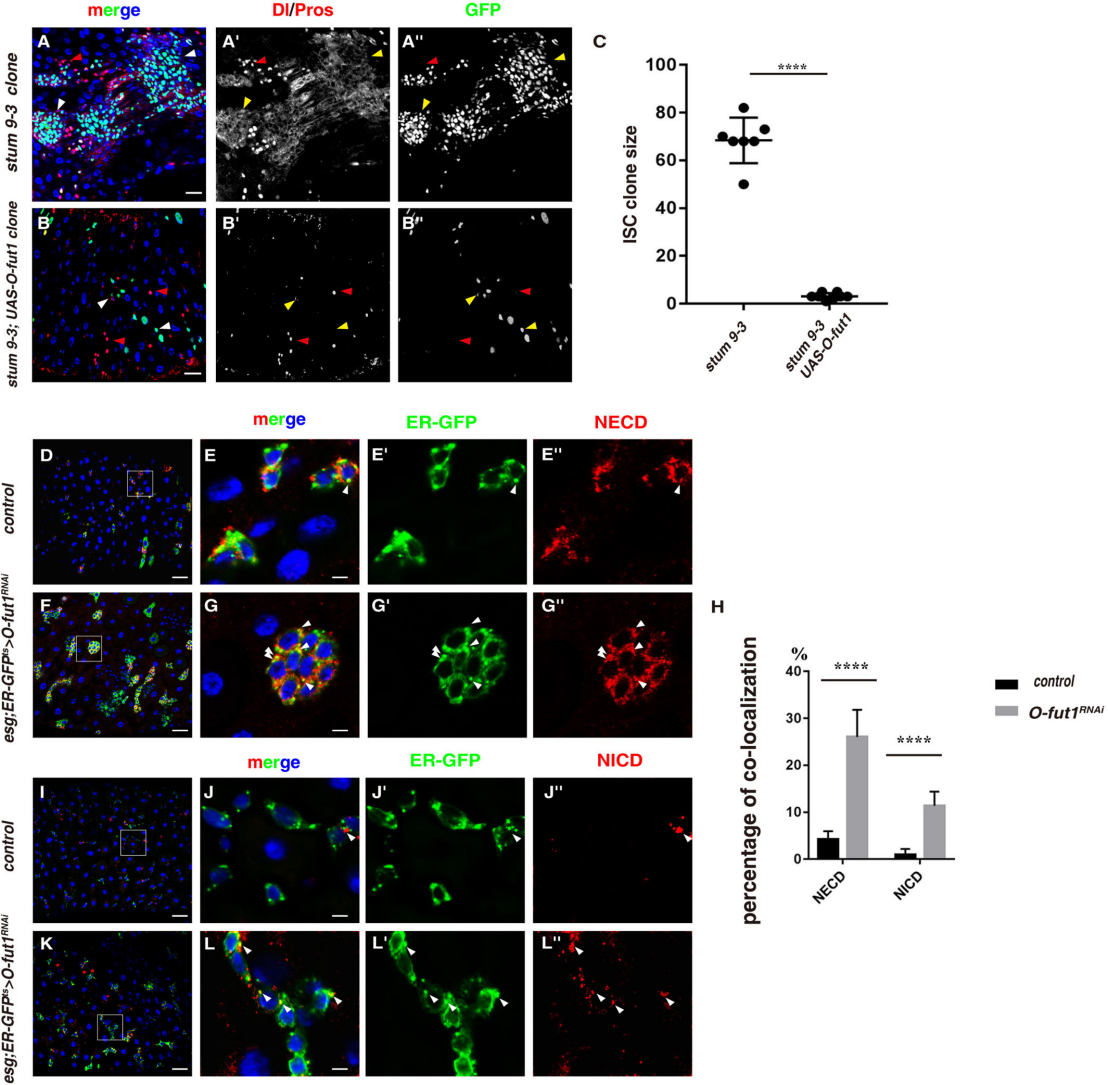
***stum 9-3* is a new allele of *O-fut1*.** (A) Dl and Pros (red) in *stum 9-3* ISC MARCM clones (white and red arrowheads). Dl/Pros and GFP channels are showed separately in black-white. (B) Expression of UAS-O-fut1 could completely rescue defects observed in *stum 9-3* ISC MARCM clones (white and red arrowheads). (C) Quantification of the size of ISC MARCM clones in different genotypes indicated. Mean±s.d. is shown. *n*=5–10 intestines. ****P<0.0001. Please note that *stum 9-3* ISC clones are deformed, preventing accurate quantification of clone size. (D–E″) NECD (red) is rarely detected in the ER (green, by KDEL-GFP) in progenitors (white arrowhead). Split channels of ER-GFP and NECD are showed separately. The boxed region in D is showed enlarged in E. (F–G″) Increased NECD puncta (red) are detected in the ER (green) in progenitors of *esg^ts^>O-fut1^RNAi^* intestines (white arrowheads). The boxed region in F is showed enlarged in G. (H) Quantification of the percentage of NECD or NICE within the ER in progenitors in control and *esg^ts^>O-fut1^RNAi^* intestines. (I–J″) NICD (red) is rarely detected in the ER (green, by KDEL-GFP) in progenitors (white arrowhead). Split channels of ER-GFP and NICD are showed separately. The boxed region in I is showed enlarged in J. (K-L″) Increased NICD puncta (red) are detected in the ER (green) in progenitors of *esg^ts^>O-fut1^RNAi^* intestines (white arrowheads). The boxed region in K is showed enlarged in L. In all panels except graphs, GFP is in green, blue indicates DAPI staining for DNA. Scale bars: 20 μm (A,B,D,F,I,K) and 5 μm (E,G,J,L). Yellow arrows correspond to the white arrows, pointing to the cells that the white arrows pointed to.

We went on to identify the gene responsible for the observed defects observed in *stum 9-3* mutant. Initial examinations showed that *stum 9-3* mutant is not allele of known genes involved in the Notch signaling pathway located on the right arm of the 2nd chromosome (2R), like *presenilin enhancer* (*pen-2*, encoding the key subunit of the γ-secretase responsible for the S3 cleavage of the Notch receptor) and *Caf1-p105* [encoding the subunit of the chromatin assembly factor 1 (CAF-1)] (Fig. S7 and data not shown). We then carried out detailed mapping of *stum 9-3* mutant using different deficiency kits for 2R and other mutants (Table S1). The lesion was finally mapped to the region of *O-fut1*, which is responsible for catalyzing the reaction that attaches fucose through an O-glycosidic linkage to a conserved serine or threonine residue found in the consensus sequence of EGF domains of the Notch receptor (Table S1) (Okajima and Irvine, 2002). To further confirm that the defects observed in *9-3* mutant is caused by mutations of *O-fut1*, we performed rescue experiments. Restoring *O-fut1* function by either over-expressing *O-fut1* or endogenous *O-fut1* under its own promoter (*O-fut1^+t3.8^*) completely rescued defects observed in *9-3* mutant (Fig. 4A–C and data not shown) (Okajima and Irvine, 2002). These data indicate that mutation of *O-fut1* in *stum 9-3* mutant is responsible for the defects observed. We further examined the nature of lesion in *O-fut1* region. We found that the lesion occurs in the 2nd coding exon of *O-fut1*, probably caused by insertion of an unknown p-element or DNA fragment (Fig. S8). We thus named this *O-fut1* allele as *O-fut1st^um9-3^*.

Depletion of *O-fut1* in progenitors resulted in identical defects as depletion of the Notch receptor (Fig. S9A–G). We further examined the consequences of depleting *O-fut1* in different types of intestinal cells using cell-type specific drivers [ISC: *DlGal4*, EB: *Gbe+Su(H)Gal4* and EE: *ProsGal4*]. Depletion of *O-fut1* in ISCs results the same defects as that of *Notch* depletion, generating clusters of ISCs and EE cells, indicating that Notch signaling is required in ISCs for their proliferation and differentiation (Fig. S9H–K). Unexpectedly, no defects were observed upon depletion of either *O-fut1* or *Notch* in EBs, in contrast to the observation that Notch signaling is activated in EBs for progeny differentiation (Fig. S9L–O). The discrepancy is likely caused by the nature of the EB driver used. The Gal4 used is directed by *Gbe+Su(H)*, the responsive element of Notch signaling activation, in EBs (Furriols and Bray, 2001; Lu and Li, 2015b). Thus genes begin to be depleted only after the expression of this EB driver, i.e. after the activation of Notch signaling in EBs. Moreover, EB cell is in a transient differentiating state. Therefore, these facts impede the detection of the requirement of genes in EBs. Interestingly, depletion of both *O-fut1* and *Notch* in EE cells produced no obvious defects (Fig. S9P–S). O-fut1 attaches fucose through an O-glycosidic linkage to the EGF domains of the Notch receptor using GDP-fucose as donor substrate in the ER (Okajima and Irvine, 2002; Okajima et al., 2005). Consistently, both NECD and NICD are accumulated in the ER upon depletion of *O-fut1* in progenitors (Fig. 4D–H). Taken together, our data show that the defects observed in *O-fut1st^um9-3^* mutant are caused by defective O-fucosylation of the full-length Notch receptor.

Two classic methods are generally deployed to identify factors involved in specific developmental process or signaling pathways: forward genetic screen and reverse genetic screen. Both methods have advantages and shortcomings. Factors involved in specific processes can be identified unbiasedly through forward genetic screen; however, the pinpoint of the lesion responsible for the defects observed is often tedious and time consuming, especially when the genetic background of the mutant is not clean, or the required deficiency (*Df*) lines are not available, or some of the *Df* lines are not correctly annotated, and it will be quite disappointing to find out that the identified mutants with interesting phenotype are new alleles of known genes after tedious mapping. The mapping of *stum 9-3* mutant is a good example for these drawbacks as we found that the genetic background of the p-element line obtained is not clean and carries multiple lethal hits, and some *Df* lines are not correctly annotated (Table S1). The genes responsible for specific processes can be easily identified from reverse genetic screens; however, the off-target effects of RNAi (RNA interference), especially for library generated by dsRNA (double strand RNA), must be carefully excluded. Thus, a combination of different experiments should be carried out to confirm that the identified gene is responsible for the defects observed.

## MATERIALS AND METHODS

### Fly lines and cultures

Flies were maintained on standard media at 25°C. Crosses were raised at 18°C in humidity controlled incubators, or as otherwise noted. Flies hatched in 18°C incubators (2–3 days old) were picked and transferred to 29°C incubator, unless otherwise specified. Flies were transferred to new vials with fresh food every day, and dissected at time points specified in the text. In all experiments, only the female posterior midgut was analyzed. Information for alleles and transgenes used in this study can be found either in FlyBase or as noted: *stum^MB01421^* (BL23736), *stum^204^* BL58774), *stum^4487^* (BL58775), *stumGal4* (BL58776 and BL58776), *Cyo, 2XTb* (gift from A. Zhu) (BL36336), *UAS-GFP-stum* (BL58778) (Desai et al., 2014), *pen-2^MI02639^* (BL36019), *UAS-Notch^FL^* (BL26820), *UAS-NICD* (Cooper and Bray, 2000), *Ote^+t5.9^* (*OteP*, gift from Chen DH) (Jiang et al., 2008), *Df(2R)* deficiency kit collections (gift from J. Pastor and from Bloomington), *O-fut1^SH2260^* (BL51666), *O-fut1^+t3.8^* (BL44244), *UAS-O-fut1* (BL9376) (Okajima and Irvine, 2002), *esgGal4, UAS-GFP, tubGal80^ts^* (*esg^ts^*, gift from N. Perrimon)*, DlGal4, UAS-GFP, tubGal80^ts^* (*Dl^ts^*, gift from S. Hou and R. Xi) (Zeng et al., 2010), *GBE+Su(H)Gal4, UAS-GFP, tubGal80^ts^* (*GBE+Su(H)^ts^*) (Lu and Li, 2015b), *vps26^RNAi^* (THU3819/HMS01769), *vps35^RNAi^* (THU3886/HMS01858), *Notch^RNAi^* (BL33611), *O-fut1^RNAi^* (THU2167/JF02052), *FRT19A-Notch^264-39^*, *Gbe+Su(H)-lacZ* (gift from S. Bray) (Furriols and Bray, 2001), *Caf1-p105^36^* (gift from R. Jiao) (Yu et al., 2013), *hsFlp, ActGal4, UAS-GFP*; *FRT42D-tubGal80* (for MARCM clonal analysis), *UAS- GFP.KDEL* (BL9898)*.*

### Immunostainings and fluorescence microscopy

For standard immunostaining, intestines were dissected in 1 X PBS (10 mmol/l NaH_2_PO_4_/Na_2_HPO_4_, 175 mmol/l NaCl, pH7.4), and fixed in 4% paraformaldehyde for 25 min at room temperature. Samples were rinsed, washed with 1 X PBT (0.1% Triton X-100 in 1 X PBS) and blocked in 5% horse serum in 1 X PBT for 45 min. Embryos were fixed and stained following standard protocol. Primary antibodies were added to the samples and incubated at 4°C overnight. The following primary antibodies were used: mouse mAb anti-Dl [C594.9B, 1:50, developed by S. Artavanis-Tsakonas, Developmental Studies Hybridoma Bank (DSHB)], mouse mAb anti-Prospero (MR1A, 1:100, developed by C.Q. Doe, DSHB), mouse mAb anti-Arm (N2 7A1, 1:100, developed by E. Wieschaus, DSHB), mouse mAb anti-Cut (2B10, 1:50, developed by G.M. Rubin, DSHB), mouse mAb anti-NECD (C458.2H, 1:30, developed by S. Artavanis-Tsakonas, DSHB), mouse mAb anti-NICD (C17.9C6, 1:30, developed by S. Artavanis-Tsakonas, DSHB), rabbit anti-β-glactosidase (Cappel, 1:5000), mouse anti-β-glactosidase (Cell Signaling Technology, 1:1000). The primary antibodies were detected by fluorescent-conjugated secondary antibodies from Jackson ImmunoResearch Laboratories. Secondary antibodies were incubated for 2 h at room temperature. DAPI (Sigma-Aldrich, 0.1 μg/ml) was added after secondary antibody staining. The samples were mounted in mounting medium (70% glycerol containing 2.5% DABCO). All images were captured by a Zeiss LSM780 inverted confocal microscope, and were processed in Adobe Photoshop and Illustrator.

### MARCM ISC clone analysis

The clonal analyses were achieved using the MARCM system (Lee and Luo, 2001). The ISC clones were induced by heat shocking 3–5 day-old adult flies at 37°C for 60 min. The flies were maintained at 25°C incubator and transferred to new vials with fresh food every day. The sizes of the marked clones were assayed at 6–8 days after clone induction (6–8D ACI, clones from at least ten midguts for each genotype were assayed).

### Generation of *UAS-pen-2-V5* and *UAS-NEXT* transgenic lines

The genomic coding region of *pen-2* (with stop codon omitted) was cloned into the XhoI and XbaI sites of a *pUAST-cV5* vector to be fused with a V5 tag at its C-terminus. The N-terminus of Notch protein (1-160 aa, containing the signal peptide) was fused to the NEXT part of Notch protein (the product after S2 cleavage, 1714-2703 aa, deleting the extracellular domain) and was cloned into a *pUAST* vector. Transgenic flies were obtained by standard P-element-mediated germline transformation.

### Data analysis

The size of ISC clones was determined using Image-Pro Plus software from each confocal image. The number of specific cells per clone was determined manually from each confocal image. The cell size in MARCM clones was determined using Image-Pro Plus software. Confocal images of 40×lens/1.0 zoom from a defined posterior midgut region between the hindgut and the copper cells of different genotypes indicated were acquired. The number of intestines scored is indicated in the text. Statistical analysis was done using the Student's *t*-test. PEMS 3.1 software was used for s.d. analyses and Sigma Plot software for graph generation. The graphs were further modified using Adobe Photoshop and Illustrator. ****P<0.0001.

## Supplementary Material

Supplementary information
