# Supplementary material for: Identification of a new allele of O-fucosyltransferase 1 involved in Drosophila intestinal stem cell regulation
Source: Biol Open. 2021 Nov 3;10(11):bio058910. doi: 10.1242/bio.058910 (PMC8576262; doi:10.1242/bio.058910)
Supplement: Supplementary information [file biolopen-10-058910-s1.pdf]

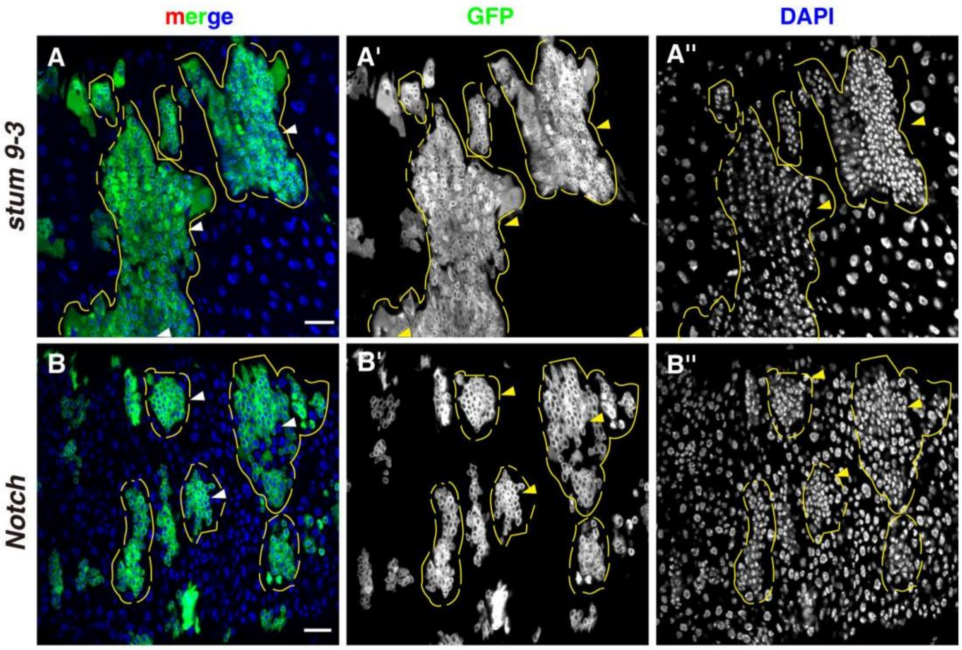

Fig. S1. *stum 9-3* clones are similar as *Notch* mutant clones.

A: *stum 9-3* ISC MARCM clones (green) (labeled with yellow dotted lines, white arrowheads). GFP and DAPI channels are showed separately in black-white. **B:** *Notch* ISC MARCM clones (green) (labeled with yellow dotted lines, white arrowheads). GFP and DAPI channels are showed separately in black-white. Scale bars: 20 µm.

Biology Open • Supplementary information

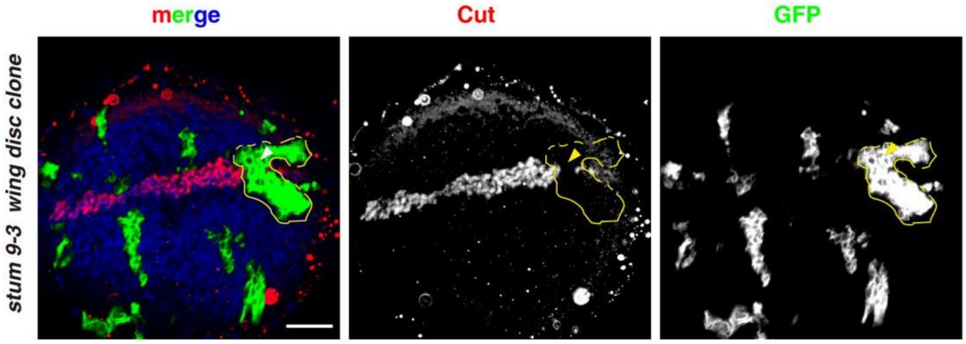

Fig. S2. ***stum 9-3*** **affects Notch signaling in the wing discs.**

Cut (red) is activated by Notch signaling in the dorsal/ventral (D/V) boundary of the 3rd instar larval wing discs. Cut signal is absent in *stum 9-3* MARCM clones (labeled with yellow dotted lines)(white arrowhead) in the D/V boundary of the wing discs. Cut and GFP channels are showed separately in black-white. Scale bar: 20 μm.

Biology Open • Supplementary information

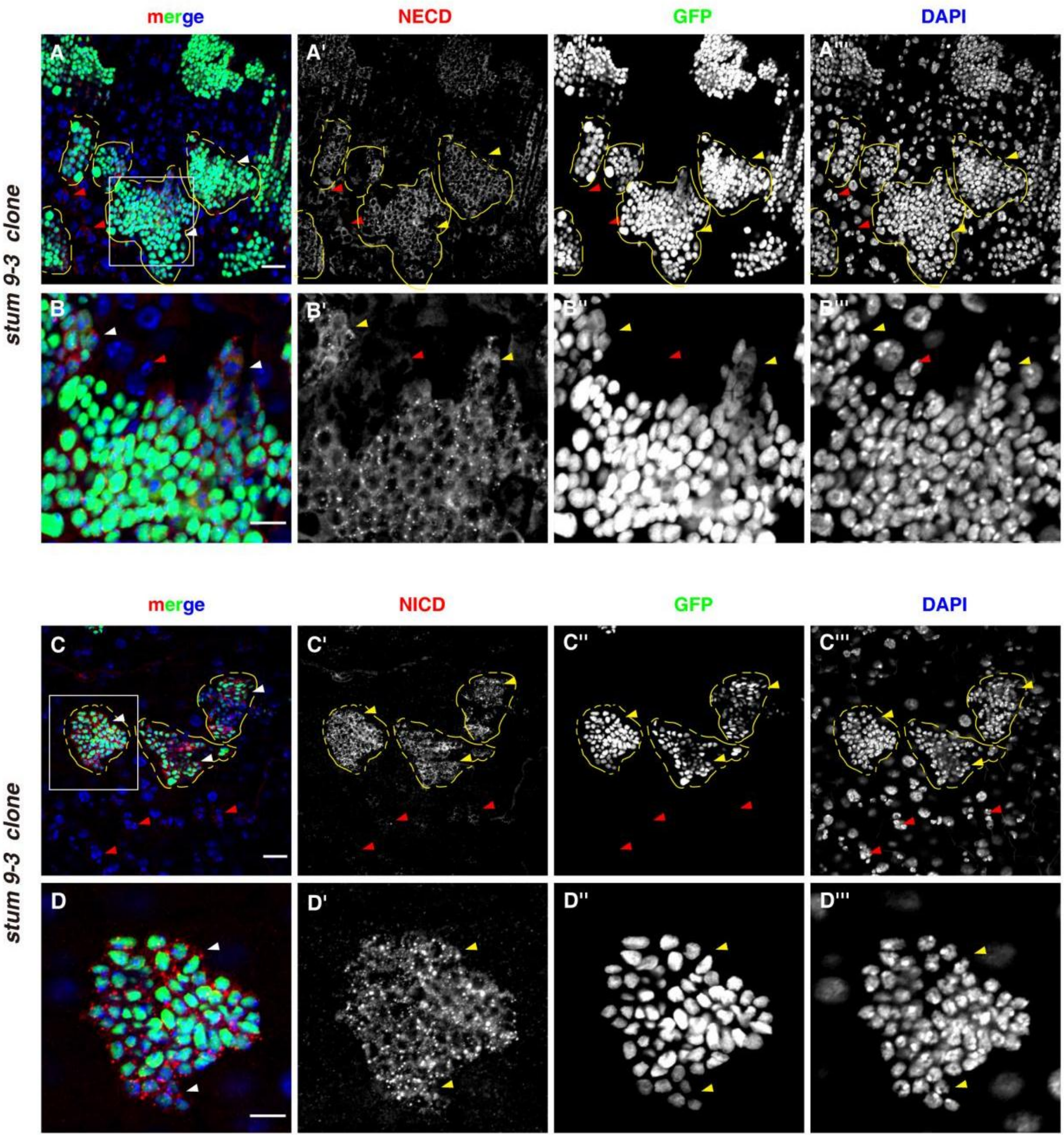

Fig. S3. Full length Notch is highly accumulated in *stum 9-3* clones.

A and **B:** NECD (red) is highly accumulated in *stum 9-3* clones (white arrowheads, with dotted yellow lines). Please note that low levels of NECD could be detected in neighboring wild type cells (red arrowheads). NECD, GFP and DAPI channels are showed separately in black-white. The boxed region in **A** is showed in **B**. **C** and **D:** NICD (red) is highly accumulated in *stum 9-3* clones (white arrowheads, with dotted yellow lines). Please note that low levels of NICD could be detected in neighboring wild type cells (red arrowheads). NICD, GFP and DAPI channels are showed separately in black-white. The boxed region in **C** is showed in **D**.

Biology Open • Supplementary information

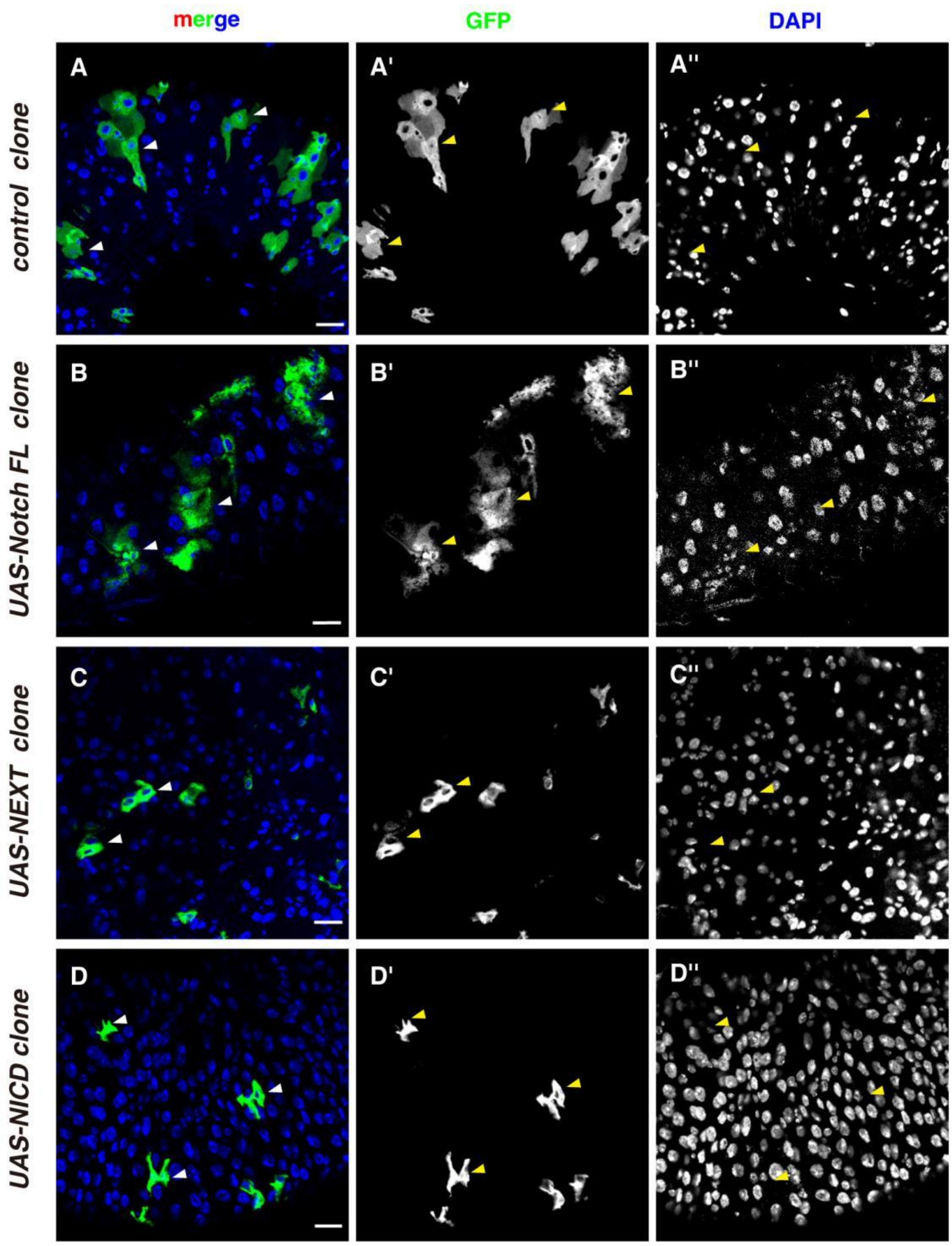

Fig. S4. ISC MARCM clones with different genotypes indicated.

A: Control MARCM clones (white arrowheads). GFP and DAPI channels are showed separately in black-white. **B:** *UAS-N^{fl}* ISC MARCM clones (white arrowheads). **C:** *UAS-NEXT* ISC MARCM clones (white arrowheads). **D:** *UAS-NICD* ISC MARCM clones (white arrowheads). GDP is in green, blue indicates DAPI staining for DNA. Scale bars: 20 µm.

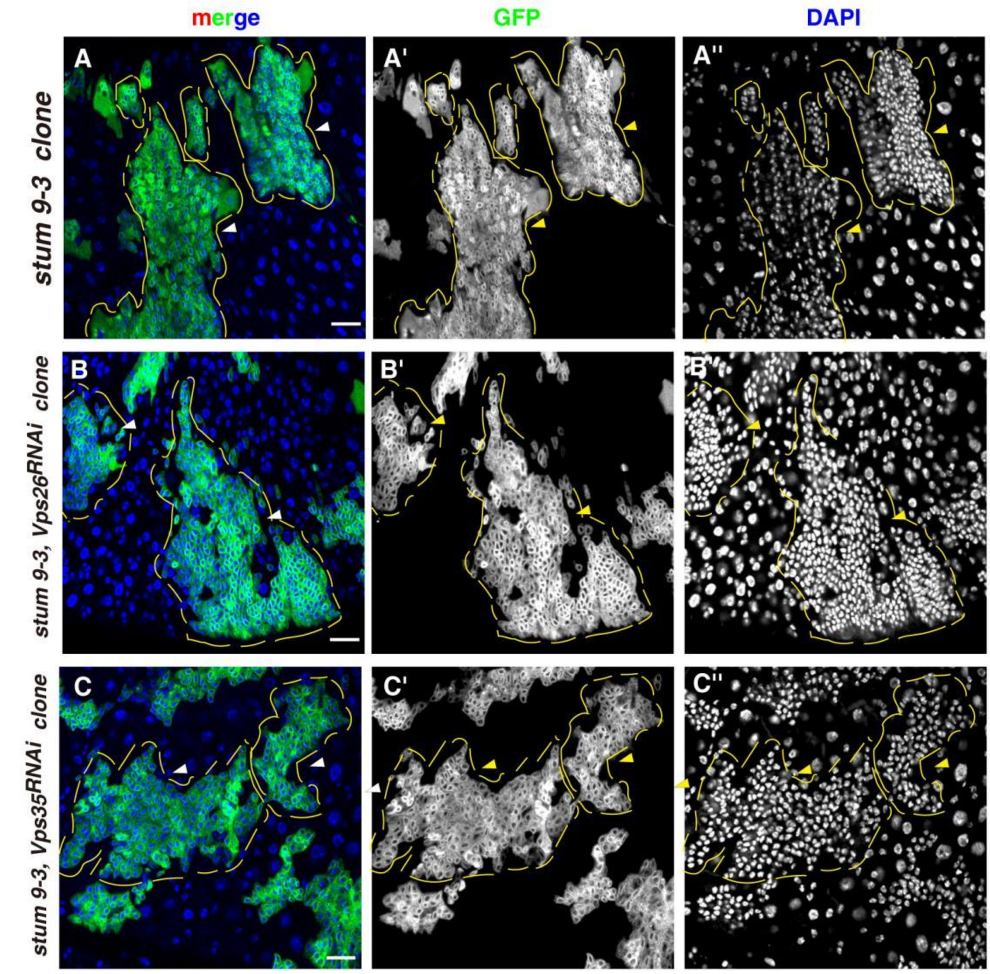

Fig. S5. The retromer complex is not involved in s*tum 9-3* mutant.

A: *stum 9-3* ISC MARCM clones (green) (labeled with yellow dotted lines, white arrowheads). GFP and DAPI channels are showed separately in black-white. **B** and **C:** Knockdown of retromer complex components (**B**: *Vps26* and **C**: *Vps35*) does not rescue defects observed in *stum 9-3* ISC MARCM clones (green) (labeled with yellow dotted lines, white arrowheads). GFP is in green, blue indicates DAPI staining for DNA. Scale bars: 20 µm.

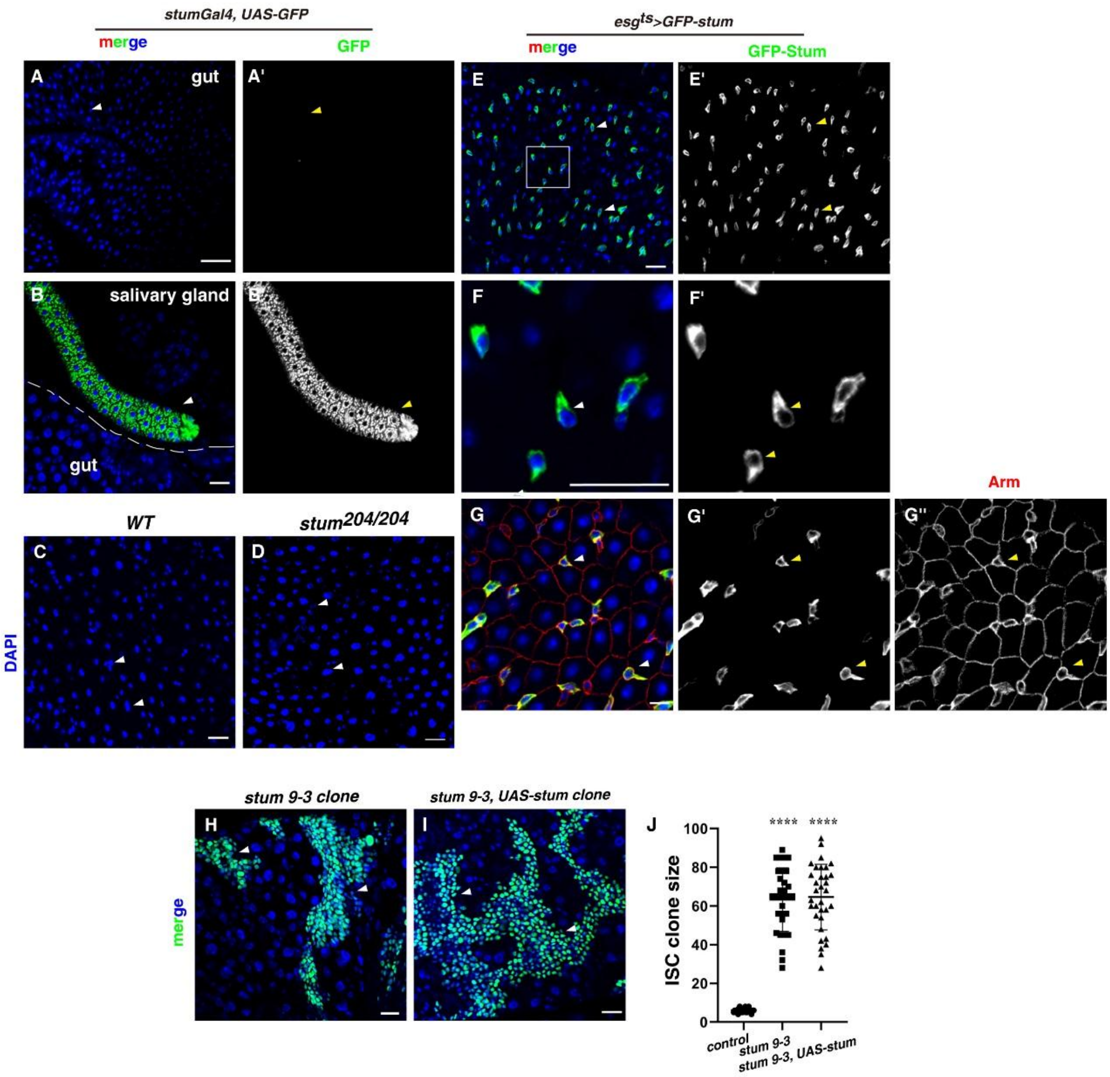

Fig. S6. ***stum 9-3*** **is not an allele of** ***stum.***

A and **B**: *stum* (by *stumGal4, UAS-GFP*, green) is not expressed in the intestine (**A**), but in the salivary gland (**B**, white arrowheads). GFP channel is showed separately in black-white. The white dotted line in **B** labels the boundary of the gut. **C** and **D:** No defects were observed in *stum* homozygous intestines (**D**), compared to the WT control (**C**, white arrowheads). **E** and **F:** esg^{ts}*>GFP-stum* intestines (*esgGal4, Gal80*^{ts}, *UAS-GFP-stum*). GFP-Stum (green, white arrowheads) is observed in the progenitors. The boxed region in **E** is showed in **F**. **G:** GFP-Stum localization in progenitors

Biology Open • Supplementary information

(white arrowheads). GFP and Arm channels are showed separately in black-white. **H** and **I:** Expression of *UAS-stum* (**I**) could not rescue the defects observed in *stum 9-3* mutant clones (**H**, white arrowheads). **J:** Quantification of the size of ISC MARCM clones indicated. Mean ± SD is shown. n = 30-35. ****P < 0.0001. Please note that the *stum 9-3* clones are highly deformed, preventing accurate quantification of the ISC clones. GFP is in green, blue indicates DAPI staining for DNA. Scale bars: 20 µm except **F** (10 µm).

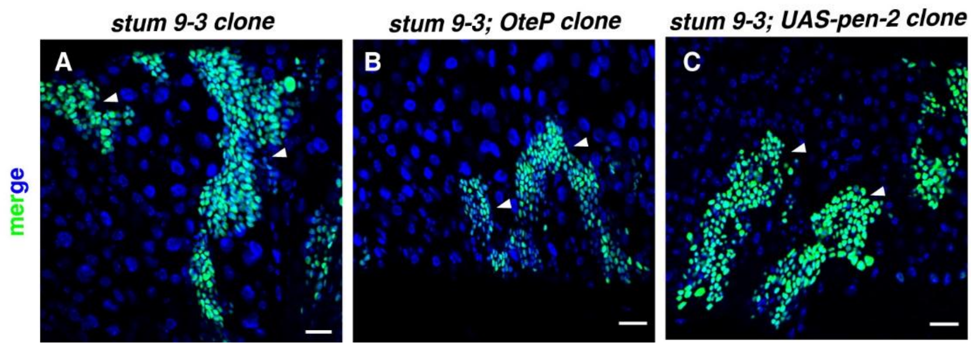

Fig. S7. s*tum 9-3* is not a *pen-2* mutant.

A: *stum 9-3* ISC MARCM clones (green) (white arrowheads). **B** and **C:** Restoring *pen-2* function (**B:** *OteP* and **C:** *UAS-pen-2*) does not rescue defects observed in *stum 9-3* ISC MARCM clones (green) (white arrowheads). GFP is in green, blue indicates DAPI staining for DNA. Scale bars: 20 µm.

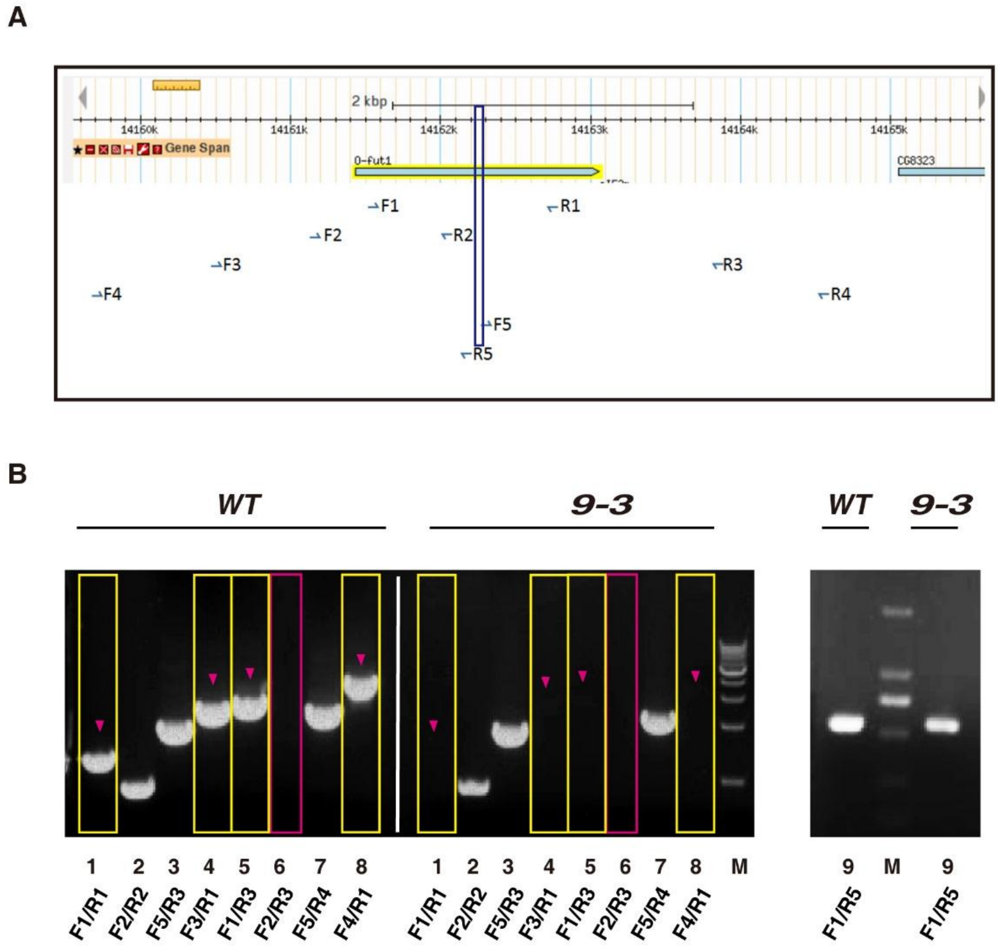

Fig. S8. Characterization of the nature of *stum 9-3* mutation.

A: Schematic cartoon of the *O-fut1* genomic region and the position of primers designed to detect the nature of *stum 9-3* mutation. The blue rectangle indicates the lesion of *stum 9-3* in *O-fut1* region. **B:** The electrophoresis of PCR products from wild type and homozygous *stum 9-3* genomic DNA. The combination of primer pairs are indicated. The yellow rectangles indicate that a product can be detected from WT, but the corresponding product can not be detected from homozygous *stum 9-3*. The pink rectangle shows that there is no product even from WT, indicating that there is something wrong. M: DNA marker.

Biology Open • Supplementary information

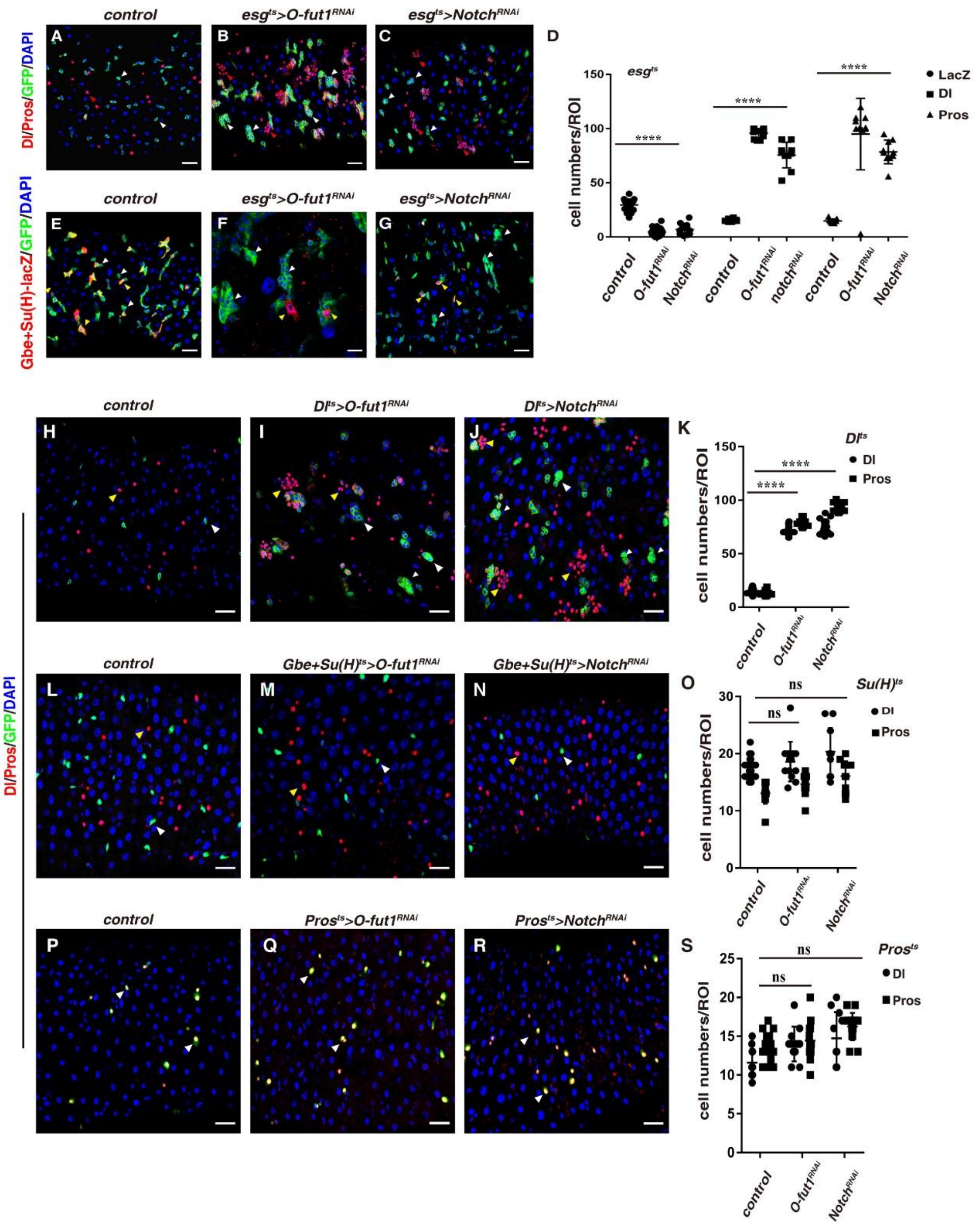

Fig. S9. *O-fut1* is involved in Notch signaling for ISC proliferation and differentiation.

A: *esg*$^{+}$ cells (green, white arrowheads) and EE cells (Pros in red, red arrowhead) in control flies at 29°C for 7 days. Dl and Pros in red. **B:** The numbers of *esg*$^{+}$ cells (green, white arrowheads) and EE cells (Pros in red, red arrowheads) in *esg*^{ts}*>O-fut1*^{RNAi} flies are dramatically increased at 29°C for 7 days. **C:** The numbers

Biology Open • Supplementary information

of *esg*$^{+}$ cells (green, white arrowheads) and EE cells (Pros in red, red arrowheads) in *esg*^{ts}>*Notch*^{RNAi} flies are significantly increased at 29°C for 7 days. **D:** Quantification of the number of *Dl*$^{+}$, Gbe+Su(H)-lacZ and EE (Pros+) cells in different genotypes indicated. Mean ± SD is shown. n = 10-15 intestines. ****P < 0.0001. **E:** *esg*$^{+}$ cells (green, white arrowheads) and EBs (Gbe+Su(H)-lacZ in red, yellow arrowheads) in control flies at 29°C for 7 days. **F** and **G:** The number of *esg*$^{+}$ cells (green, white arrowheads) in *esg*^{ts}>*O-fut1*^{RNAi} (**F**) and *esg*^{ts}>*Notch*^{RNAi} (**G**) flies is dramatically increased at 29°C for 7 days, but the number of Gbe+Su(H)-lacZ+ cells is significantly reduced (yellow arrowheads). **H:** ISCs (green by *Dl*^{ts}>*GFP*, white arrowhead) and EE cells (Pros in red, red arrowhead) in control flies at 29°C for 7 days. Dl and Pros in red. **I** and **J**: The numbers of ISCs (white arrowheads) and EEs (Pros in red, yellow arrowheads) in *Dl*^{ts}>*O-fut1*^{RNAi} (**I**) and *Dl*^{ts}>*Notch*^{RNAi} (**J**) flies are dramatically increased at 29°C for 7 days. **K:** Quantification of the number of ISCs and EE (Pros+) cells in different genotypes indicated. Mean ± SD is shown. n = 10-15 intestines. ****P < 0.0001. **L:** EBs (green by *Gbe+Su(H)*^{ts}>*GFP*, white arrowhead) and EE cells (Pros in red) in control flies at 29°C for 7 days. Dl and Pros in red. **M** and **N**: The numbers of EBs (white arrowheads) and EEs (yellow arrowheads) in *Gbe+Su(H)*^{ts}>*O-fut1*^{RNAi} (**M**) and *Gbe+Su(H)*^{ts}>*Notch*^{RNAi} (**N**) flies are not significantly changed at 29°C for 7 days. **O:** Quantification of the number of ISCs and EE (Pros+) cells in different genotypes indicated. Mean ± SD is shown. n = 10-15 intestines. ns: not significant. **P:** EEs (green by *Pros*^{ts}>*GFP* and Pros in red, white arowheads) in control flies at 29°C for 7 days. Dl and Pros in red. **Q** and **R**: The

Biology Open • Supplementary information

number of EEs (yellow arrowheads) in *Pros*^{ts}*>O-fut1*^{RNAi} (**Q**) and *Pros*^{ts}*>Notch*^{RNAi} (**R**) flies is not significantly changed at 29°C for 7 days. **S:** Quantification of the number of ISCs and EE (Pros+) cells in different genotypes indicated. Mean ± SD is shown. *n* = 10-15 intestines. ns: not significant. GFP is in green, blue indicates DAPI staining for DNA. Scale bars: 20 µm.

Supplemental Experimental Procedures

PCR primers to detect *O-fut1* (*stum 9-3*) mutation:

F1：CAGAATGCAGTGGCTCAAAA

R1：CTCGTGCACGTTTGTATGCT

F2：AGCTGGTCTCGTTCGACTGT

R2：TGGATCGTTCTTCTCCTGCT

F3：AGGCAAAATCGGCAGTGTTT

R3：CTTCGTCGGAACAGATGCAG

F4：TTGCTTTTCACAGGACACGG

R4：CACAAGATCATCGGCGTCTG

F5：CCGGCTAGTTTTCCTGTTCA

R5：AGTGGCGCATAGAACTCTGA

Biology Open • Supplementary information
